# Autoimmune Neurological Conditions Associated With Zika Virus Infection

**DOI:** 10.3389/fnmol.2018.00116

**Published:** 2018-04-11

**Authors:** Yeny Acosta-Ampudia, Diana M. Monsalve, Luis F. Castillo-Medina, Yhojan Rodríguez, Yovana Pacheco, Susan Halstead, Hugh J. Willison, Juan-Manuel Anaya, Carolina Ramírez-Santana

**Affiliations:** ^1^Center for Autoimmune Diseases Research, School of Medicine and Health Sciences, Universidad del Rosario, Bogota, Colombia; ^2^Institute of Infection, Immunity and Inflammation, University of Glasgow, Glasgow, United Kingdom

**Keywords:** Zika virus, autoimmunity, Guillain-Barré syndrome, Transverse myelitis, molecular mimicry

## Abstract

Zika virus (ZIKV) is an emerging flavivirus rapidly spreading throughout the tropical Americas. *Aedes* mosquitoes is the principal way of transmission of the virus to humans. ZIKV can be spread by transplacental, perinatal, and body fluids. ZIKV infection is often asymptomatic and those with symptoms present minor illness after 3 to 12 days of incubation, characterized by a mild and self-limiting disease with low-grade fever, conjunctivitis, widespread pruritic maculopapular rash, arthralgia and myalgia. ZIKV has been linked to a number of central and peripheral nervous system injuries such as Guillain-Barré syndrome (GBS), transverse myelitis (TM), meningoencephalitis, ophthalmological manifestations, and other neurological complications. Nevertheless, mechanisms of host-pathogen neuro-immune interactions remain incompletely elucidated. This review provides a critical discussion about the possible mechanisms underlying the development of autoimmune neurological conditions associated with Zika virus infection.

## Introduction

Zika virus (ZIKV) from the genus *Flavivirus* is an emerging mosquito-borne pathogen part of the Spondweni serocomplex. ZIKV was first isolated in 1947 from the serum of a febrile sentinel monkey in the Zika forest in Uganda, east Africa (Dick et al., 1952). The first human infection was reported in Nigeria in 1954, and later, in 1962 a ZIKV strain was isolated from an adult male in Uganda (Simpson, 1964). In 2007, a large human outbreak outside of Africa was reported on Yap Islands in the Federated States of Micronesia (Hayes, 2009). The next outbreak of ZIKV occurred in French Polynesia in 2013 and 2014 and was unprecedented, with an estimated 28,000 cases of ZIKV infection (Cao-Lormeau et al., 2014). Subsequent ZIKV outbreaks occurred on other Pacific Islands including the Cook Islands, New Caledonia, and Easter Island (Musso et al., 2014). ZIKV spread rapidly throughout the Americas after its initial appearance in northeastern Brazil in May 2015, possibly by infected travelers (Campos et al., 2015). Since then, transmission of ZIKV has been reported throughout South America, Central America, the Caribbean, Mexico, and the USA. In August 2016, the PAHO reported 578,148 suspected cases of ZIKV in 45 countries and territories in the Americas (PAHO WHO, 2016).

**Figure 1:**
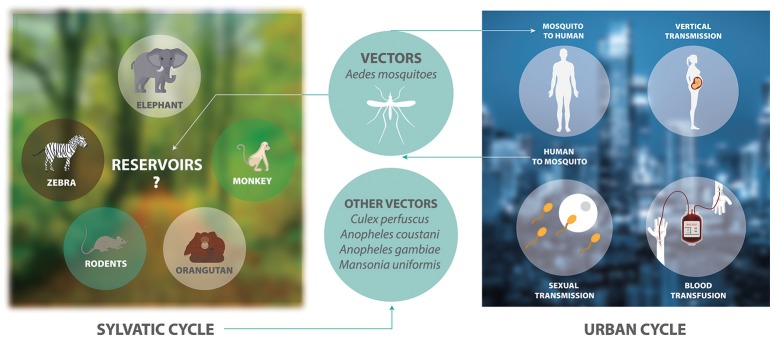
The transmission cycle of ZIKV. The sylvatic cycle involves the maintenance of ZIKV between non-human primates and arboreal mosquitoes in forests. There is only serological evidence showing that elephants, zebras, rodents, and orangutans are possible reservoirs of ZIKV. The urban cycle involves the transmission of ZIKV between humans and mosquitoes in urban areas.

ZIKV is an arthropod-borne virus with two transmission cycles (Figure 1). The sylvatic cycle is tangled in the maintenance of ZIKV between non-human primates and arboreal mosquitoes in forests, whereas in the urban cycle is implicated in the transmission of ZIKV from humans to urban mosquitoes (Weaver et al., 2016). ZIKV is transmitted mainly by *Aedes* species mosquitoes including *Aedes africanus, Aedes luteocephalus, Aedes vittatus, Aedes furcifer, Aedes apicoargenteus, Aedes hensilli, Aedes aegypti*, and *Aedes albopictus*. Mosquitoes acquire the virus via blood meal, and host it throughout their life-span without adverse effects (Suzuki et al., 2017; Zhao et al., 2018). Thus, ZIKV is transmitted to human through the bite of female infected *Aedes* species mosquito, most commonly *A. aegypti* and *A. albopictus*. These two species of mosquitoes generated epidemic risk due to their dynamic adaptation to urban environments, their capacity to survive to extreme environmental conditions or to be dispersed passively by humans, their ability to tolerate moderate climates and keep sylvatic niches, together with the urbanization and migration (Saiz et al., 2017). On the other hand, species that belong to genera other than *Aedes*, including *Culex perfuscus, Anopheles coustani, Anopheles gambiae*, and *Mansonia uniformis* were found to be infected with ZIKV in Africa, proving that these mosquitoes must have fed on a viremic vertebrate (Saiz et al., 2017). Moreover, anti-ZIKV antibodies were detected in wild mammals in Senegal in 1967–1968 (Brès, 1970). In Indonesia, anti-ZIKV antibodies were detected in ducks, goats, cows, horses, bats, and carabaos (Olson et al., 1983). In 1983, Darwish and collaborators reported anti-ZIKV antibodies in rodents, sheep and goats in Pakistan (Darwish et al., 1983). In Malaysia, samples collected between 1996 and 1997 from wild and semi-captive orangutans were positive for anti-ZIKV antibodies (Wolfe et al., 2001). The detection of these antibodies were the first findings of probable ZIKV infection in rodents and domestic animals. However, the natural history of this virus must be investigated in more detail.

Other transmission routes are sexual activities, perinatal transmission from mother to fetus, and blood transfusion (Musso et al., 2014). ZIKV RNA has been detected in semen and female genital tract samples (Saiz et al., 2017). Also, many studies have showed evidence of sexual transmission (Moreira et al., 2017). Studies in rhesus and cynomolgus macaques indicate that transmission of ZIKV by sexual intercourse is a mechanism of virus maintenance in the absence of mosquito transmission and could increase the probability of spread of ZIKV in regions where this virus is not present (Haddow et al., 2017). These different ways of transmission of this virus make it difficult to develop control strategies against ZIKV.

ZIKV infection can be symptomatic in 18–57% of cases; thus, it may be asymptomatic in up to 80% of cases. It causes a minor, self-limiting disease with an incubation period of maximum 10 days (Ahmad et al., 2016). Viremia is generally seen within 3–4 days after onset of symptoms. Symptomatic patients may develop fever and symptoms typical of arboviral infections, such as rash, joint pain, conjunctivitis, headache, and myalgia (Ahmad et al., 2016). These relatively mild symptoms last a few days. However, ZIKV appears to be neuroinvasive (6.5 × 10^7^ viral RNA copies/mg of brain tissue; Mlakar et al., 2016) and has been linked to numerous neurological complications including congenital brain abnormalities (Gerardin et al., 2017), infant microcephaly (Johansson et al., 2016), Guillain-Barré syndrome (GBS) (Oehler et al., 2014; Pinto-Diaz et al., 2017), and meningoencephalitis (Carteaux et al., 2016; Table 1).

**Table 1:** Neurological manifestations and ZIKV infection.

**Geographic location**	**Publication date**	**Study type**	**Number of cases**	**Gender**	**Neurological symptoms**	**Cerebrospinal fluid results**	**Neuroimaging**	**ZIKV symptoms**	**ZIKV confirmation**	**Observations**	**Diagnosis**	**References**
Martinique	2016	Case series	2	No details	Convulsive seizures, GCS 9	Normal	MRI normal	Fever, headache and arthralgia	RT-PCR in plasma, CSF and urine	EEG normal. Other possible viral infections were discarded	Encephalopathy	Roze et al., 2016
				No details	Mental confusion, speech disorder		MRI Leukoaraiosis	Headache, conjunctivitis, myalgia and arthralgia		EEG focal activity	Encephalopathy right facial palsy	
Pacific Islands	2016	Case report	1	Male	Fever 39.1°C, GCS 6, hemiplegia of the left side, paresis of the right upper limb. Mechanical ventilation was needed.	Suggestive of meningitis	MRI Suggestive of Meningoencephalitis	Asymptomatic	RT-PCR in CSF Viral culture from CSF on a Vero cell line	Neurologic condition improved without specific treatment. However, a left arm weakness (4/5) persisted after he was discharged from the ICU	Meningoencephalitis	Carteaux et al., 2016
Brazil	2017	Case report	1	Male	Fever, headache, malaise, transitory left-sided hemiplegia and generalized seizures.	High protein levels Lymphocytic pleocytosis Second sample was suggestive of meningitis	MRI low cerebral blood flow with cytotoxic cortical edema surrounded by vasogenic edema	No details	RT-PCR of the CSF Brain biopsy was consistent with immunohistochemistry, immunofluorescence, and electron microscopy findings of ZIKV infection	Immunosuppressed. Patient died.	Meningitis	Schwartzmann et al., 2017
Dominican Republic	2016	Case report	1	Female	Asthenia, bilateral leg weakness. Attention and cognitive impairment in neuropsychological tests.	Sample 1 Normal Sample 2 Lymphocytic pleocytosis	MRI normal	Fever, rash, headache and conjunctivitis	RT-PCR in Serum, CSF, saliva, vaginal secretion and urine IgM serum	IGIV was administered	Encephalitis	Nicastri et al., 2016
Brazil	2016	Case report	1	Female	Leg weakness, speech disorder and confusion. Mechanical ventilation was required	Mild lymphocytic pleocytosis. High proteins	CT Brain scan showed massive brain swelling	Rash, arthralgia	RT-PCR in urine was positive and negative in serum	Dengue virus IgM titers were negative. As well as Herpes Simplex virus 1 and 2 in CSF. Patient died	Encephalitis	Soares et al., 2016
Puerto Rico	2016	Surveillance report	1	No details	Encephalitis	No details	No details	–	RT-PCR (+)	No details	Encephalitis	Dirlikov et al., 2016
Colombia	2017	Case control	3	2 Males	All patients presented altered mental status and fever In 2/3, generalized or partial seizures and meningeal signs were observed 2 were admitted to the ICU and required mechanical ventilation	In only 1/2 of these two patients was found pleocytosis High proteins	MRI normal	–	IgM negative and IgG positive in Serum samples (ELISA)	Patients had previous history of Dengue and Chikungunya virus infection.	Encephalitis	Anaya et al., 2017
Colombia	2017	Case control	3	Females	These patients presented a decrease or loss of movement in facial muscles and sensory disturbances	No details	No details	No details	IgM negative and IgG positive in Serum samples (ELISA)	Patients recovered without neurological sequelae	Peripheral facial palsy	Anaya et al., 2017
Colombia	2017	Case control	1	Female	Abnormal gait associated with urinary retention. Decreased reflexes in limbs, weakness and a decrease in temperature sensation in neck and abdomen	No details	No details	Fever, rash, arthralgia, conjunctivitis and diarrhea.	IgM negative and IgG positive in Serum samples (ELISA)	–	Thoraco-lumbosacral myelopathy	Anaya et al., 2017
Colombia	2017	Case control	6	4 Male	They all presented a monophasic disease Hyper-reflexia, and a defined sensory level Autonomic signs were observed such as arrhythmia (2/6), urinary retention (3/6), ileus (3/6), and blood pressure lability (1). 2 were admitted in ICU	In 3/6 patients were performed lumbar puncture 2/3 showed pleocytosis	MRI 4/6 In 3/4 patients was possible to determine vertebral segment involvement	–	IgM negative and IgG positive in Serum samples (ELISA)	Presence of autoantibodies was evaluated IgG anti—aquaporin 4 and anti-Ro antibodies (negative results) 1 patient had positive anti-phospholipid antibodies	Transverse myelitis	Anaya et al., 2017
Leeward Islands (French Caribbean Islands)	2016	Case report	1	Female	Left arm weakness, lower back pain, paraesthesia on the left side of her body. She presented loss of temperature sensation below the T2 dermatome on the left and T4 on the right. As well as bladder dysfunction.	CSF was normal	Brain MRI was normal Spinal MRI evidenced cervical and thoracic spinal cord lesions	Headache, left arm pain and conjunctival hyperaemia	RT-PCR in serum, urine and CSF	PCR in CSF was negative for viral and bacterial agents. Aquaporin-A antibodies were also negative.	Acute myelitis	Mecharles et al., 2016
Colombia	2016	Case report	1	Male	Pelvic pain followed by urinary retention, lower limb weakness that resulted in paraplegia. Loss of sensation that compromised T6-T7 dermatomes	CSF high proteins and lymphocytic pleocytosis	Brain and thoracolumbar CT scan were normal MRI suggestive of transverse myelitis	Conjunctival hyperaemia, fever and arthralgia	RT-PCR in serum	Patient underwent Plasmapheresis therapy IgM was negative for Dengue virus and Chikungunya	Transverse myelitis	Palacios et al., 2016
Brazil	2017	Observational cohort	3	1 Male	Back pain (1), lower limb weakness (2), sensory deficits (2), ataxia (1) Both patients required ICU	–	–	Rash (2), conjunctivitis (1), fever (2), arthralgia (1), headache (1)	2/3 RT-PCR in serum	A female patient had idiopathic transverse myelitis	Transverse myelitis	da Silva et al., 2017
Brazil	2016	Case report	3 infants	1 Male	One mother presented rash and arthralgias in 1st trimester. All infants had unilateral ocular findings (gross macular pigment mottling) and foveal reflex loss. 1 infant presented neuroretinal atrophy	No details	CT scans evidenced cerebral calcifications	–	No test were performed	–	Microcephaly + cerebral calcifications	Ventura et al., 2016
Brazil	2017	Case report	1 stillborn	Male	One mother on 13th week of gestation presented fever, myalgia, arthralgia, retroocular pain and conjunctivitis	No details	Microcephaly, ventriculomegaly, calcifications and cerebral atrophy on 29th week ultrasonography	–	Indirect immnuofluorescence, RT-PCR (+) and electron microscopy	Fetal autopsy was performed at 32 weeks and 6 days of gestation	Microcephaly	Strafela et al., 2017

Arboviral infections may alter the immune recognition of peripheral nerve, possibly causing the myelin and underlying axon not to be recognized as self-tissue. This would make these structures a target for abnormal autoimmune responses. This article provides updated information about the potential mechanisms underlying the development of autoimmune neurological conditions associated with ZIKV infection.

## Biology of ZIKV

Phylogenetic analyses of ZIKV genomes expose the presence of two principal viral lineages, Asian, and African. Yun and collaborators performed phylogenetic analysis with the nucleotide sequences of the 29 accessible ZIKV genomes, finding the following genetic lineages: African, including MR-766 (African lineage, Uganda, 1947); and Asian, including both PRVABC-59 (Asian lineage-derived American strain, Puerto Rico, 2015) and P6-740 (Asian lineage, Malaysia, 1966; Yun et al., 2016). ZIKV involved in the outbreak in Brazil and in the Americas has been found to come from the Asian-lineage virus, which was isolated in French Polynesia between 2013 and 2014 (de Melo Freire et al., in review).

ZIKV has been classified as a member of the family *Flaviviridae*, genus *Flavivirus* with an enveloped, icosahedral virion of 40–50 nm in diameter containing the non-segmented, single-stranded, positive-sense RNA genome of 10,794 nucleotides in length (White et al., 2016). This genome has two non-coding regions at the 5′ and 3′ end of the genome and a single long open reading frame, encoding a polyprotein that is cleaved into capsid (C), envelope (E), membrane precursor (prM), and seven non-structural proteins (NS1, NS2A, NS2B, NS3, NS4A, NS4B, and NS5; Kuno and Chang, 2007). The C protein is basic and complexes with the viral RNA in the nucleocapsid, whereas the outer membrane of the virion is a lipid bilayer containing the viral membrane protein (M) and E protein. The M protein is expressed as a glycosylated prM, and the E protein is responsible for viral entry and represents a key determinant for viral pathogenesis (Neal, 2014). E glycosylation is important for ZIKV infection of mammalian and mosquito hosts (Fontes-Garfias et al., 2017). NS1 protein is associated with the evasion of the immune system of the host and appears to be involved in viral replication along with NS4A. NS2A is involved in virus assembly and NS2B acts as a cofactor for NS3 protease domain. NS3 protein is involved in viral replication and in the polyprotein processing. NS4A and NS4B protein is involved in the inhibition of Akt-mammalian target of rapamycin (mTOR) signaling pathway. NS5 appears to be involved in suppressing the interferon (IFN) signaling, which is mediated via proteasome-dependent degradation of Signal Transducer and Activator of Transcription 2 (STAT2) (Mishra et al., 2017).

**Figure 2:**
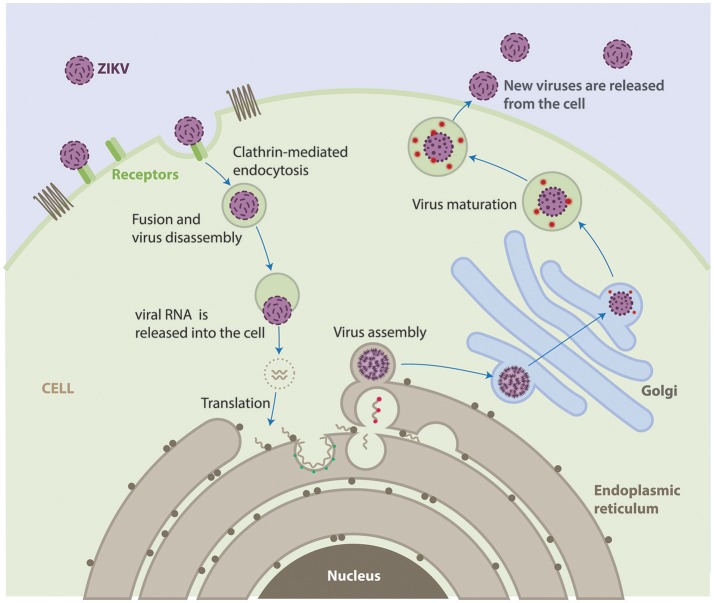
Life cycle of ZIKV. ZIKV attaches to the surface of a host cell and enters the cell by a process called endocytosis. Once deep inside the cell, the virus fuses with the endosomal membrane and it is released into the cytoplasm. The virus particle releases the viral genome. The viral RNA is translated into a single polypeptide that is cut into 10 proteins, and the viral genome is replicated. Virus assembly occurs on the surface of the endoplasmic reticulum. The immature viral particles are transported through the trans-Golgi network, where they mature and convert to their infectious form. The mature viruses are released from the cell and can go on to infect other cells.

The life cycle of ZIKV is similar to other known flaviviruses (Figure 2). Briefly, virions attach to the surface of the host cell by interactions between viral surface glycoproteins and cell surface receptors and subsequently enter the cell by receptor-mediated endocytosis and are internalized into clathrin-coated pits. Subsequently, the viral RNA is released into the cytoplasm following fusion of the viral and host membranes. The positive-sense genomic RNA is translated into a single polyprotein that is processed cotranslationally and post-translationally by cellular and viral proteases. This cleavage makes a total of three structural proteins and seven non-structural proteins. Genome replication occurs on vesicle packages, thus facilitating the assembly of the viral replication complex (Hamel et al., 2015). Virus assembly occurs on the surface of the endoplasmic reticulum, these new particles travel alongside the host secretory pathway through the trans-Golgi network, where virion maturation occurs and then is released by exocytosis (Lindenbach and Rice, 2003; Roby et al., 2015).

## Neuropathogenesis of ZIKV

**Figure 3:**
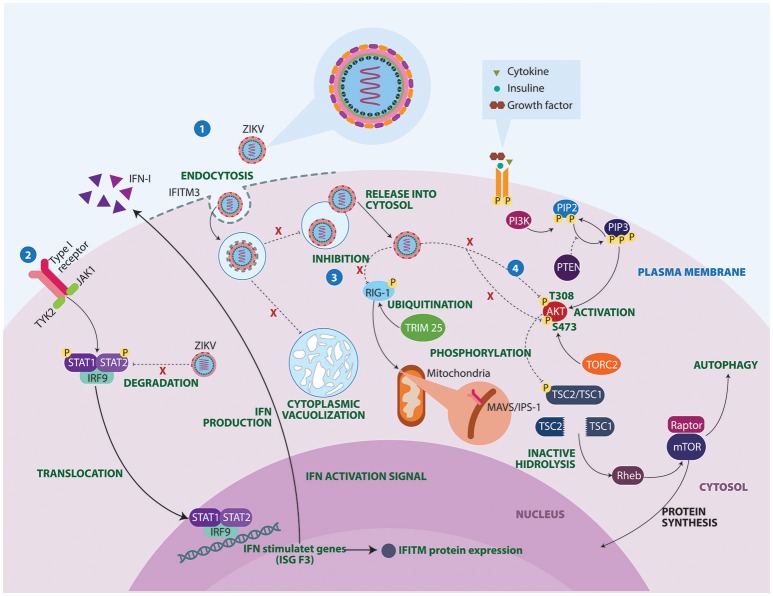
Molecular mechanisms of ZIKV underlying the neuropathogenesis. 1. IFITM3 proteins confers immunity to the ZIKV. However, failure in the expression of this transmembrane protein allows viral replication, cell-fusion and massive vacuolization. 2. ZIKV protein NS5 binds and destroys STAT2 via proteosomal degradation, impeding interferon production. 3. Activated retinoic acid-inducible gene 1 (RIG-1) receptors recognize viral components and induce an antiviral immune response. However, ZIKV manages to inhibit these sensors, conferring resistance to IFN products. 4. Lastly, ZIKV proteins NS4A and NS4B interrupt phosphorylation of AKT at two sites T308 and S473. As a result, ZIKV infection turns out to be a substantial stressor for the Akt pathway, which could have important clinical implications in brain functioning and development. IFITM3, Interferon induced transmembrane protein 3; IFN, Interferon; IRF9, Interferon regulatory factor 9; ISG F3, Interferon-stimulated gene factor 3; JAK1, Janus kinase 1; MAVS, Mitochondrial antiviral-signaling protein; mTOR, Mammalian target of rapamycin; PI3K, Phosphoinositide 3-kinase; PIP2, Phosphatidylinositol 4,5-bisphosphate; PIP3, Phosphatidylinositol (3,4,5)-trisphosphate; PTEN, Phosphatase and tensin homolog; Rheb, Ras homolog enriched in brain; STAT, Signal transducer and activator of transcription; TORC2, Transducer of CREB protein 2; TRIM 25, Tripartite motif-containing protein 25; TSC, Tuberous sclerosis; TYK2, Tyrosine Kinase 2.

The mechanisms underlying ZIKV-induced neuropathogenesis are still poorly understood. However, studies in mice and guinea pigs showed that ZIKV can replicate and affect CNS cells (Dick, 1952; Bell et al., 1971; Kumar et al., 2017). Also, recent studies have used *in vitro* technologies to elucidate mechanisms that contribute to development of autoimmune neurological alterations after Zika infection (Figure 3). Some studies have described the mechanisms by which ZIKV avoids the host IFN signaling of STAT2. During viral infection IFN-I pathways are activated, allowing the expression of hundreds of IFN-stimulated response elements. ZIKV protein NS5 binds and destroys STAT2 via proteasomal degradation, conferring viral resistance to IFN in cell cultures (Grant et al., 2016; Kumar et al., 2016).

Another potential mechanism linking ZIKV infection to neurological disease concerns the inhibition of RIG-I molecules (Donald et al., 2016). RIG-I-like receptors (RLRs) are viral RNA sensors required to initiate an innate immune response through type I IFN production (Oshiumi et al., 2016). These recognition receptors are able to induce a proinflammatory cytokine state. This may explain why in acute phases, a Th1, Th2, Th9, and Th17 response is observed in patients with ZIKV infection (Tappe et al., 2016). A closer look at the activation of cytoplasmic retinoic acid inducible gene RLRs, shows that they need to undergo a post-translational modification process facilitated by Tripartite motif-containing protein 25 ubiquitin ligase (Gack et al., 2007). A defect in these non-specific defense mechanisms could facilitate GBS manifestations following ZIKV infection.

ZIKV infects a broad range of neural cells including neural stem cell, astrocytes, oligodendrocyte precursor cells, and microglia (Retallack et al., 2016; Cumberworth et al., 2017). The ability of the virus to induce implosive cell death in fibroblasts and astrocytes is another interesting mechanism observed in ZIKV pathogenesis. Imaging studies have demonstrated ZIKV infection triggers cytopathic effect on infected cells in which ZIKV-infected cells undergo morphological changes with massive vacuolization followed by implosion (Monel et al., 2017). IFN induced transmembrane family proteins are restriction factors implicated in the prevention of the viral cell-fusion of multiple viruses. Failure in the expression of these transmembrane proteins is associated with an increase of ZIKV-induced cell death (Savidis et al., 2016).

In assessing ZIKV proteins, a recent study suggests that expression of ZIKV viral proteins is responsible for cytopathic effects including cell-cycle disturbance, inhibition of cell proliferation, and cell death in host cells. For instance, the expression of prM protein resulted in cell-cycle G1 accumulation, whereas cell-cycle G2/M accumulation is observed in membrane-anchored capsid, M protein, E protein, and NS4A protein. Thus, Li and collaborators demonstrated that NS4A, expressed individually in a fusion yeast model, triggers inhibition of proliferation, cell hypertrophy, cell-cycle dysregulation, and cellular oxidative stress leading to cell death through Tor1 and type 2A phosphatase activator Tip41 proteins (Li et al., 2017). However, ZIKV proteins NS4A and NS4B impede phosphorylation of Akt at those specific sites in the mTOR pathway (Liang et al., 2016). Moreover, the presence of neurologic syndromes possibly are related to the existence of high cytokine levels, as it is found in ZIKV-infected neural crest cells, which in some way, may induce cytotoxicity *in vitro* (Bayless et al., 2016). In this context, ZIKV infection turns out to be a substantial stressor for the Akt pathway, which could have important clinical implications in brain functioning and development. In addition, dysregulation in the autophagy might induce myelin injury similar to the one observed in multiple sclerosis patients, in which augmented expression of *Atg5* gene was associated with immune-mediated myelin injury in experimental autoimmune encephalomyelitis (Alirezaei et al., 2009).

Few approaches have been proposed to determine the relationship between viral RNA persistence and the presence of neurologic syndromes. The frequency of ZIKV RNA and the lag time term differs between fluids. A preliminary study demonstrated that viral RNA clearance may take ~14–80 days in serum; 8–39 days in urine and 34–125 days in semen samples (Paz-Bailey et al., 2017). Lozier and collaborators demonstrated that time-to-loss of ZIKV RNA in serum was longer in adults than in children, and conjunctivitis was associated with detection of ZIKV RNA in semen (Lozier et al., 2017). These data raise the possibility that ZIKV may co-exist in different anatomic regions, such as lymph nodes and neural cell compartments.

It appears that viral survival in the central nervous system is associated with activation of mTOR, pro-inflammatory, and anti-apoptotic pathways (Aid et al., 2017). This phenomenon may be linked to neurological manifestations caused by ZIKV, even days following viral clearance from peripheral blood.

## Guillain-Barré syndrome

GBS is a neurological disorder characterized by an aberrant activation of the immune system that results in the damage of peripheral nervous system (Sejvar et al., 2011; Willison et al., 2016). Patients with GBS develop a rapidly ascending neuromuscular paralysis followed by a loss in sensitivity and pain perception. Although the pathogenesis of this syndrome is not fully understood, most cases have in common a recent respiratory or gastrointestinal infection (Tam et al., 2007; Mahecha et al., 2017). Microorganisms such as *Campylobacter jejuni, Mycoplasma pneumonia*, Cytomegalovirus, Epstein-Barr virus, *Haemophilus influenza*, Hepatitis E, as well as human immunodeficiency virus, and ZIKV have been implicated in triggering the onset of GBS (Brannagan and Zhou, 2003; Monsalve et al., 2017; Rodríguez et al., 2018a). Furthermore, GBS cases associated with vaccines have also been reported (Israeli et al., 2012; Sejvar, 2014).

Acute inflammatory demyelinating polyneuropathy (AIDP), acute motor axonal neuropathy (AMAN), and acute motor sensory axonal neuropathy (AMSAN) are clinical variants of GBS, principally defined through electrophysiological studies, underpinned by pathological findings. The underlying driver of GBS is believed to be due to a loss of immunological tolerance to self-antigens (Shoenfeld et al., 1996). There is evidence that the antibodies bind to epitopes on the outer myelin surface producing complement activation and myelin destruction previous macrophage invasion (Hafer-Macko et al., 1996). These macrophages release cytokines and free radicals, invade myelin sheaths and act as scavengers in order to remove myelin debris (Yuki and Hartung, 2012). Damage to myelin sheaths, nodes of Ranvier, and nerve axons can disrupt nodal Nav channel clusters and subsequently cause nerve conduction failure. In AMAN, antibodies are directed against ganglioside components of the motor nerves and nodes of Ranvier, whereas in AMSAN, antibodies affect both motor and sensory fibers (Hughes and Cornblath, 2005). IgG antibodies against GM1, GD1a, GalNAc-GD1a, and GM1b are found in patients with AMAN and AMSAN (Dalakas, 2015). Furthermore, the production of different ganglioside antibodies is associated with certain clinical manifestations including Bickerstaff brainstem encephalitis and Miller Fisher syndrome (Ito et al., 2008; Dagklis et al., 2016).

The molecular mechanisms of ZIKV underlying the pathogenesis of GBS are still not at all understood. However, multiple host-virus interactions have been proposed to induce disease. Some of these are focused on molecular mimicry, antibody dependent enhancement of ZIKV infection, T-cell immunoreactivity, humoral immunity, and viral neurotropism for neuron and glial cells (Anaya et al., 2016; Munoz et al., 2016).

One widely considered hypothesis implicated in this disease is best described by molecular mimicry. According to Lucchese et al., ZIKV polyproteins share peptides with human proteins that, when altered, are associated with GBS. These analyzes suggested that many of the shared peptides may be endowed with immunological potential. In other words, ZIKV infection could cross-react with some brain proteins and other molecules that might contribute to the ZIKV-associated neuropathologic sequelae (Lucchese and Kanduc, 2016). In the case of ZIKV-associated GBS, high titers of ZIKV antibodies could lead to cross-reactivity between component of ZIKV and neuronal membrane gangliosides. In a case-control study, Cao-Lormeau et al., found that patients with ZIKV infection and GBS had some evidence of anti-glycolipid antibody activity against GA1, GM2, GD1a, and GD1b antigens (Cao-Lormeau et al., 2016). This suggested the possible role of molecular mimicry in ZIKV-GBS pathogenesis.

Sera from patients diagnosed with GBS which tested positive for ZIKV infection in Cucuta, Colombia (Anaya et al., 2017), between June 2015 and 2016 were also screened for the presence of anti-glycolipid IgG and IgM antibodies. The results of this study demonstrated the absence of such antibodies at greater frequency than non-neurological, post-ZIKV infected group (unpublished data). This is unusual given that 11/42 (26.2%) patients in this cohort were diagnosed with the axonal (AMAN/AMSAN) subtype of the disease and IgG anti-ganglioside antibodies are frequently associated with the axonal variant of GBS. One explanation for this may be the extended lag between neurological onset and serum collection (median time 100 days, range 36–242 days) in this acute phase disease.

Moreover, Lucchese and Kanduc found that more than 500 immunogenic epitopes are shared by the virus and human neural proteins, when related to axonal neuropathies and myelin disorders (Lucchese and Kanduc, 2016). The proteins identified as the targets of antibodies to high probability ZIKV mimic epitopes, including pro-neuropeptide Y, neuron navigator 2, neurogenic differentiation factor 4, brain-derived neurotrophic factor, and neurexins, are proteins with diverse roles in neurologic function and in embryonic development (Homan et al., in review). These homologies highlight the potential complexity of GBS pathogenesis mediated by ZIKV.

Lastly, antibody-dependent enhancement of Zika could result in severe neurological complications (Vatti et al., 2017). This may be triggered by a previous immunological response, in which circulating antibodies bind to the virus but it is not able to neutralize infection. Rather, these antibodies increase the number of infected cells and virus replication (Flipse et al., 2013). Under laboratory conditions, the phenomenon of antibody-dependent enhancement is observed in ZIKV experiments (Dejnirattisai et al., 2016; Paul et al., 2016). Interestingly, a previous infection with *M. pneumoniae* was observed to be a high risk for developing GBS in patients infected with ZIKV (Anaya et al., 2017). However, the role of previous infection with *M. pneumoniae* in the development of GBS associated with ZIKV deserves further investigation.

## Transverse myelitis

Transverse myelitis (TM) is also considered an immune-mediated syndrome. TM causes neural injury to the spinal cord with concurrent acute or subacute dysfunction, resulting in varying clinical manifestations as described below (Krishnan et al., 2004; Cree and Wingerchuk, 2005). The incidence of TM ranges between 0.134 and 0.460 new cases per 100,000 habitants per year (Berman et al., 1981; Bhat et al., 2010). Although TM can occur at any age, it has been observed a bimodal peak between the ages of 10 and 19 years and 30 and 39 years (Berman et al., 1981; Christensen et al., 1990; Jeffery et al., 1993). Furthermore, it has been observed that, females have a higher risk of developing TM than males (Beh et al., 2013).

Clinically, patients with TM present signs and symptoms associated to motor, sensory and autonomic nerves dysfunction (Cree and Wingerchuk, 2005). Concerning weakness, this is described as rapidly progressive beginning in the legs and infrequently progresses to the arms. The most common sensory level in adults is the mid-thoracic region, nonetheless children may have a higher frequency of cervical sensory level (Pidcock et al., 2007). In relation to autonomic nerve involvement, autonomic dysfunction may be a common complication of TM. This can take place in the acute or chronic phases of TM and occurs mainly in lesions above the upper thoracic segments (Beh et al., 2013). Additionally, associated to the acute spinal cord lesion, it could cause a neurogenic shock as a severe complication (Krassioukov et al., 2007).

Myelopathies can be subdivided into compressive and non-compressive causes. Between the latter, TM is one of the main important ones. Etiologies for TM can be classified as disease-associated TM when patient shows standard criteria for known causes or idiopathic TM when an extensive search fails to determine the exact cause (Barnes et al., 2002). Among the causes of disease-associated TM are paraneoplastic syndromes and parainfectious causes acquired (de Seze et al., 2001; Jacob and Weinshenker, 2008). On the other hand, demyelinating disorders as multiple sclerosis, neuromyelitis optica, and acute disseminated encephalomyelitis have been strongly associated with TM (Borchers and Gershwin, 2012). Additionally, other systemic autoimmune diseases such as systemic lupus erythematosus (Mok et al., 1998), antiphospholipid syndrome (Dar et al., 2015), and Sjögren's syndrome (Alhomoud et al., 2009) could be included in the list of causes of TM.

**Figure 4:**
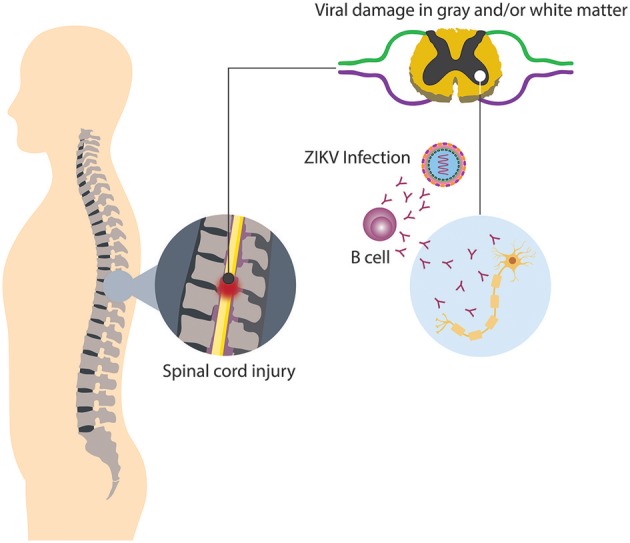
Neurologic damage by ZIKV. Immune regulatory mechanisms fail, thus culminating in the breakdown of self-tolerance, resulting in immune-mediated attack directed against both viral and self-antigens.

It has been noted that in approximately half of the cases of TM is parainfectious, i.e., the neurologic injury related with TM may be associated to direct microbial infection, or indirect infection followed by a systemic response, thus inducing neural injury (Bhat et al., 2010; Beh et al., 2013). Among the causative agents of parainfectious TM are bacteria, parasites, fungi, and viruses. Concerning viral infection and TM, recently ZIKV appears to be a new triggering agent of the disease, since in some countries where outbreaks have occurred, associated cases of TM have been reported as a neurological complication distinct from GBS (Mecharles et al., 2016; Palacios et al., 2016; Anaya et al., 2017). Up to know, it has been difficult to determine if parainfectious TM, in this case triggered by ZIKV, is produced by direct viral invasion or a consequence of inflammatory mechanisms induced by the infection (Figure 4; Beh et al., 2013). In relation to viral invasion, the virus can access to an immune restricted site, evading the immune surveillance present in other organs. Such a mechanism may also explain the limited inflammation at a focal region of the spinal cord present in TM patients (Kerr and Ayetey, 2002).

Although the infectious agent in these cases may be present within the central nervous system, other immune-mediated mechanisms, such as molecular mimicry and superantigen-mediated disease, require only peripheral immune activation and may to be part of the pathophysiology of TM (Kaplin et al., 2005). Even though, it remains unclear the mechanisms by which ZIKV can generate TM, as in GBS, molecular mimicry could be a plausible one. In this case, the human neural tissue contains numerous subtypes of ganglioside moieties within their plasma membranes, similar to different microorganisms, generating an immune response and later development of autoantibodies. The development of abnormal antibodies probably activate other components of the immune system and/or recruit additional cellular components to the spinal cord as is observed in neuromyelitis optica or multiple sclerosis, two diseases strongly related with TM (Lin et al., 2017; Prineas and Parratt, 2017; Yoshikura et al., 2017).

The production of autoantibodies seen in TM patients suggests that a direct and selective injury of neurons containing antigens that cross-react with antibodies directed against infectious pathogens may occur (Kaplin et al., 2005). Another possible link between ZIKV and TM may be the activation of lymphocytes by viral superantigens. It is possible that some ZIKV peptides not identified can activate T lymphocytes in a different way compared with conventional antigens that activate a more aggressive cellular response.

Immune disruption in cellular and humoral response described before could be associated with monocytes and lymphocytes infiltration into segments of the spinal cord and perivascular spaces and an invariable astroglial and microglial activation observed y pathological specimens (Katz and Ropper, 2000; Krishnan et al., 2004). Moreover, in postinfectious TM, the presence of white and gray matter inflammatory changes, associated with demyelination and axonal injury has been described. On the other hand, two different immune responses during acute phase and subacute TM phases have been elucidated. During the acute phases, infiltration of CD4+ and CD8+ lymphocytes in the central compartment of the cord, along with an increased presence of monocytes, is quite prominent. Furthermore, in subacute phases, prominent monocyte and phagocytic-macrophage infiltration is detected (Krishnan et al., 2004). In addition, the high prevalence of different autoantibodies in TM patients proposes polyclonal imbalance of the immune system. It may also be that some autoantibodies initiate a direct and selective injury of neurons containing antigens that cross-react with antibodies against pathogens. These confirm that TM is an immune mediated disorder that involves cellular responses and feasibly humoral factors that wound compartments of the spinal cord (Krishnan et al., 2004).

## Autonomic system involvement

Dysautonomia has been observed in up to 76% of patients with GBS during ZIKV infection (Anaya et al., 2017). This percentage is certainly higher than that one found in patients with GBS associated with other etiologies (González et al., 2016). This phenomenon may be due to an additive effect of ZIKV on the GBS development, or an indirect autonomic dysfunction affecting the organs innervated by the autonomic system without affecting the autonomic nerves, as has been observed in animal models of West Nile Virus (WNV) infection (Wang et al., 2011; Maramattom et al., 2014). WNV is another arbovirus which may induce autonomic dysfunction in humans regardless of the presence of GBS (Leis and Stokic, 2012). Therefore, based on the above mentioned data we underwent a case-control study aimed to evaluate autonomic symptoms in ZIKV infected patients, by using the composite autonomic symptom scale 31 (COMPASS-31) (Rodriguez et al., 2018b). Patients with previous ZIKV infection had significantly higher COMPASS-31 score than controls, regardless of age and sex. The main drivers for the higher scores where orthostatic intolerance, secretomotor, and bladder symptoms (Rodriguez et al., 2018b). Several pathogenic mechanisms have been proposed to explain autonomic dysfunction due to a viral infection (Carod-Artal, 2018), including invasion of the central nervous system and the direct viral, toxin-mediated or immune-mediated association of the peripheral and autonomic nervous system (Carod-Artal, 2018). Using a neuronal culture model from murine, it was determined that ZIKV persistently and effectively infects sensory neurons of the trigeminal and dorsal root ganglia (Swartwout et al., 2017). Autonomic neurons that innervate these regions were not tolerant for ZIKV infection. Nevertheless, ZIKV infection of satellite glial cells that frame and support sensory and autonomic neurons in peripheral ganglia lead to in their destruction (Swartwout et al., 2017). Thus, if autonomic nerve damage during the acute ZIKV infection in the absence of other neurological manifestations is confirmed, the mechanisms should be fully investigated, and early diagnosis will become fundamental for the suitable treatment of autonomic dysfunction.

## Conclusions and perspectives

The recent ZIKV outbreaks have triggered the occurrence of neurological manifestations likely associated to this arbovirus. Molecular mimicry between glycolipids and surface molecules of infectious agents has been proposed as a possible pathogenic mechanism of autoimmune diseases, this hypothesis is supported in GBS. Also, most of the cases of TM appear to be parainfectious. Further studies aimed at elucidating the underlying pathogenic mechanisms responsible of neurologic injuries associated with ZIKV infection are needed, as well as assays designed to identify the targets of the autoimmune response and viral cross-reactivity. It is important to note that other factors combined with ZIKV infection may be the cause of these neurological disorders, for this reason more genetic, environmental and immunological research are needed. Finally, ZIKV surveillance and monitoring programs should be implemented to control outbreaks of ZIKV in the future.

## Author contributions

YA-A, DM, SH, HW, J-MA, and CR-S: organized and revised the manuscript. YR, YP, and LC-M: equally contributed to the writing of this review. All authors approved the final manuscript.

### Conflict of interest statement

The authors declare that the research was conducted in the absence of any commercial or financial relationships that could be construed as a potential conflict of interest.

## Abbreviations

AIDP: Acute inflammatory demyelinating polyneuropathy
AMAN: Acute motor axonal neuropathy
AMSAN: Acute motor sensory axonal neuropathy
C: Capsid protein
E: Envelope protein
GBS: Guillain-Barré Syndrome
IFN: Interferon
M: Membrane protein
mTOR: Mammalian target of rapamycin
NS: Non-structural protein
prM: Precursor of membrane
RLRs: RIG-I like receptors
STAT2: Signal transducer and activator of transcription 2
TM: Transverse myelitis
WNV: West Nile Virus
ZIKV: Zika virus.
